# Standardized Outcomes Measures in Physical Therapy Practice for Treatment and Rehabilitation of Cerebral PALSY: A Systematic Review

**DOI:** 10.3390/jpm11070604

**Published:** 2021-06-26

**Authors:** Maria Dolores Apolo-Arenas, Aline Ferreira de Araújo Jerônimo, Alejandro Caña-Pino, Orlando Fernandes, Joana Alegrete, Jose Alberto Parraca

**Affiliations:** 1 Departamento Terapéutica Médico Quirúrgica, Facultad de Medicina, Universidad de Extremadura, 06006 Badajoz, Spain; mdapolo@unex.es (M.D.A.-A.); alejandrocp@unex.es (A.C.-P.); 2 Departamento de Desporto e Saúde, Escola de Saúde e Desenvolvimento Humano, Universidade de Évora, 7004-516 Évora, Portugal; alinea.fje@gmail.com (A.F.d.A.J.); orlandoj@uevora.pt (O.F.); joana.alegrete@fa.uevora.pt (J.A.); 3 Comprehensive Health Research Centre (CHRC), Universidade de Évora, 7004-516 Évora, Portugal

**Keywords:** cerebral palsy, physical therapy, outcome assessment

## Abstract

Cerebral palsy (CP) treatment includes physical therapy and various complementary therapies to the standard clinical treatment. However, there are not many reviews that focus on the methods used and evaluation procedures. This study aims to analyze which tools are most suitable for the evaluation and methodology of patients with CP treated with physical therapy. Following the PRISMA statement, through a PICOS strategy, PubMed/MEDLINE, Web of Science (WOS), Scopus, Science Direct, and Scielo were searched with the following terms: cerebral palsy AND (physical therapy modalities OR therapeutics) AND outcome assessment. The methodological quality of the RCTs was assessed with the Evidence Project risk of bias tool. Thirty-seven RCTs and six RCT protocols, comprising 1359 participants with different types of CP: spastic hemiplegia/paresis, spastic diplegia/paresis, and spastic CP, met the inclusion criteria, uncovering 21 variables measured through 77 different instruments and several interventions. The therapies most widely used in CP are gaming or technology-assisted therapies, aerobic training, hippotherapy, music therapy, gait training, and aquatic exercises. This study provides an overview of what the authors used in the neurorehabilitation field through procedure evaluation and checking the technological advance that began to be used.

## 1. Introduction

Cerebral palsy (CP) definition has been evolving throughout time. According to Rosembaum [1], “Cerebral palsy (CP) describes a group of permanent disorders of the development of movement and posture, causing activity limitation, that are attributed to nonprogressive disturbances that occurred in the developing fetal or infant brain. The motor disorders of CP are often accompanied by disturbances of sensation, perception, cognition, communication, and behavior, by epilepsy, and by secondary musculoskeletal problems”.

According to Surveillance of Cerebral Palsy in Europe (SCPE), CP can be classified per motor impairment, covering spastic, dyskinetic, ataxic, nonclassifiable types, and distribution (unilateral or bilateral, depending on the involved brain side) [2]. Despite prenatal and other unknown causes representing most cases of CP, premature birth constitutes the principal risk factor; when accounting for this, 10–15% of all CPs are postnatal [3]. The diagnosis starts with a medical history check to evaluate abnormal findings congruent with CP’s symptoms. It is posteriorly confirmed through specific evaluation methods such as neuroimaging, standardized neurological, and standardized motor assessments [4]. As seen in CP, impairments in motor function are often associated with communicative, cognitive, and perceptive problems that negatively influence educational [5] and vocational development [6].

The most important element of CP treatment is multifaceted improvement. The major role in this improvement is played by systematic and comprehensive motor rehabilitation, individually tailored to the patient [2]. The literature shows the positive effects of conventional physical therapy methodologies, and other multimodal complementary modalities approached from physical therapy, such as music therapy, hydrotherapy, or animal-assisted therapy (e.g., equine-assisted therapy) [7]. Although each patient presents a variety of functional disorders and treatment is multifactorial, the results of each treatment may vary [8]. Therefore, the assessment of the therapy outcomes is critical [8]; once accomplished, it validates the therapy or allows for replacement with one more reliable, making the treatment more effective in the long term [9].

Despite the availability of several instruments for CP’s outcome analysis that reflect the variety of functional disorders and specific restrictions, the literature focuses on the benefits of interventions in CP [10]. Additionally, instruments and procedures used to characterize CP are challenging to choose because there are ample options with particular outcomes, which are not always present in the patients and sometimes require specific training. As a result, there is a need for systematic knowledge about the methods and procedures for evaluating the outcome of CP treatment. An incisive knowledge through the available method and procedures on CP assessment outcomes may contribute to the design of investigation that is focused on functional diversity patients, thus facilitating the choice of method and procedure.

Therefore, this study analyzes which tools are the most suitable to measure outcomes in CP patients treated with physical therapy, providing an overview of evaluation procedures used in different physical therapy modalities and determining if technological advance has begun to be implemented in the process.

## 2. Methods

This study followed the Preferred Reporting Items for Systematic Reviews and Meta-Analyses (PRISMA) statement.

### 2.1. Eligibility Criteria

The PICOS strategy was defined, in which (P) refers to people from 1 year old up to 50 years old, of any sex or ethnicity with a diagnosis of CP. Abbreviation (I) corresponds to physical therapy or any technique within physical therapy modalities; (C) refers to a group with no intervention, comparing different interventions, or the same group before and after the intervention; (O) corresponds to evaluation methods applied to analyze the outcomes related to physical capacities, functionality, and quality of life after intervention; and (S) indicates randomized control trials studies (RCT). The inclusion criteria were (a) articles observing evaluation methods applied for analysis of benefits obtained with physical therapy; (b) studies analyzing the results of an intervention plan and reporting the number of sessions; (c) available full-text articles in Spanish, English, or Portuguese; and (d) the last five-year time coverage, from 2016 to September 2020. Exclusion criteria were studies (a) about masticatory function, (b) with drug treatments or invasive procedures, (c) with individuals having CP associated with other neurological dysfunction, (d) with surgical interventions, and (e) with interventions focused on orthoses. 

### 2.2. Information Sources and Search Strategy

A structured search strategy was developed using the Medical Subject Headings (MeSH) vocabulary. According to the strategy, in September 2020, we searched the following electronic databases: PubMed/MEDLINE, Web of Science (WOS), Scopus, Science Direct, and Scielo. The MeSH terms used were cerebral palsy AND (physical therapy modalities OR therapeutics) AND outcome assessment. In addition, for the Pubmed database, we applied two filters: “randomized control trial” and “5 years”. In the other databases selected, we applied a time-related filter (last five years); this time interval for eligible studies was defined with the intent to provide the most recent literature on the topic. Nonspecific filters to select randomized control were available, although none were used.

### 2.3. Study Selection and Data Extraction

**Figure 1:**
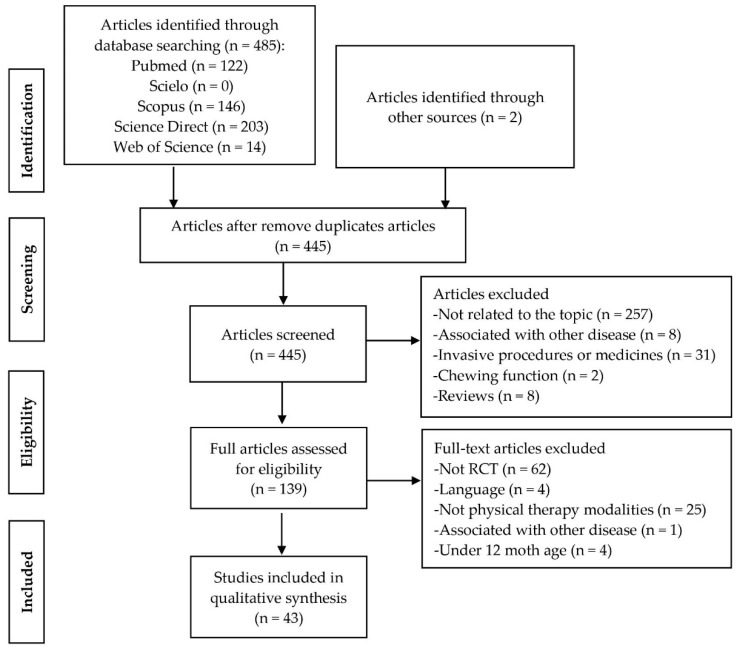
Flowchart of study selection in electronic databases.

To guarantee the established eligibility criteria, two reviewers (A.F.A.J. and J.A.P.) performed separately the first screen of titles and abstracts of the studies on electronic databases, which investigated all full text of potentially relevant articles. A third reviewer (M.D.A.A.) was consulted to solve any inclusion/exclusion disagreements. Figure 1 shows all the details for the eligibility criteria used for the selection of the articles. Afterward, data extraction was performed by two reviewers together (A.F.A.J. and M.D.A.A.) and the following data were extracted from the selected studies: name of the first author, year of publication, country of origin, aims of the investigation, study population (sample size and age), type of CP, intervention, and main results.

### 2.4. Risk of Bias

Methodological quality of the randomized control trials was assessed with the Evidence Project risk of bias tool [11]. This instrument comprises eight items of three different domains, and responses can vary as Yes (rated as 1) or No (rated as 0) to create a total score (0–8). A higher result represents a lower bias risk.

## 3. Results

The search strategy in the different electronic databases revealed 485 articles, of which 40 studies were duplicates and 43 met the inclusion criteria (Figure 1). From the selected studies, 37 were RCTs and 6 were RCTs protocols. We included the 6 RCT protocols once the methodology outcome measures were well established. Supplementary Files 1 and 2 summarize the characteristics of the selected studies.

### 3.1. Participants

In the 43 studies, 1359 participants with different types of CP formed the analyzed population group. Samples from the studies varied between 102 and 6 subjects. Some authors did not reference palsy typography, referring only to CP [12,13,14,15,16,17]. From the other studies, 12 focused on spastic hemiplegia/paresis, 9 focused on spastic diplegia/paresis, 7 on just spastic CP, 7 investigated unilateral and bilateral spastic CP and 2 studies included subjects with all types of CP [18,19]. The participants were also classified according to the Gross Motor Function Classification System (GMFCS). Most parts of the studies selected participants who were at levels I to III of impairment (*n* = 23). In addition, eight studies classified the sample according to the Manual Ability Classification System (MACS) and included participants with mild levels of limitations (I–III). Only nine studies included individuals in level III to V of GMFCS, while three articles did not classify the sample according to GMFCS or any other scale. Regarding age, the range of analyzed subjects was from nineteen months to twenty-eight years old; however, 95.2% of studies (*n* = 40) focused their attention on investigating children with CP.

### 3.2. Outcomes Measures

#### 3.2.1. Variables

The study revealed many instruments to measure CP’s outcomes that we organized according to the International Classification of Functioning, Disability, and Health (ICF) domains (Table 1). The most cited variables were related to gross motor function and hand and arm motor skills, both variables in the activity and participation component of ICF (Table 1). Another variable well cited by the authors was dynamic balance and was related to involuntary movement reaction functions. Personal factors, such as health-related quality of life, spasticity in muscle tone functions, gait pattern, and aerobic capacity in exercise tolerance, were also frequently investigated by the authors (Table 1). Some authors assessed functioning and disability using the ICF for children and youth, among others tests that analyze multiple dimensions related to mobility, domestic life, and life habits in general (Table 2).

#### 3.2.2. Instruments

We identified 77 instruments to measure the supra cited variables. The instruments more cited were (a) Time Up and Go (TUG), which assesses dynamic balance in involuntary movement reaction function; (b) 6 min walk test (6MWT), which assesses the estimated aerobic capacity in exercise tolerance; (c) Gross Motor Function Measurement (GMFM) scale, version 66, which evaluates changes in gross motor function; (d) Modified Ashworth Scale (MAS), an instrument for spasticity related to muscle tone function; (e) hand-held dynamometer that assesses muscle strength; and (f) Pediatric Evaluation of Disability Inventory (PEDI) to measure functional status in children with CP (Table 1).

The therapeutic interventions in CP analyzed with the assessment instruments presented were gaming or technology-assisted therapies, which were the most common type of intervention used by the authors [15,20,21,22,23,24,25,26,27,28,29,30,31,32,33], followed by electrotherapy management [12,34,35,36,37,38,39,40,41], and strength training [42,43,44,45,46]. Although less frequently, the studies investigated aerobic training [14,15,16,17], hippotherapy [18,47], music therapy [48,49], gait training [50], and aquatic exercise [51]. Two studies focused their attention on evaluating the effectiveness of home-based programs, both involving technology advice: rehabilitation robot [52] and side-alternating whole-body vibration equipment [13]. A few authors investigated areas such as manual therapy [53], respiratory physical therapy [54], and alternative therapies [55]. Finally, [16] analyzed the effect of segmental training in gross motor function, whereas [19] focused their attention on sports-specific fundamental movement skills training.

### 3.3. Risk of Bias

The mean score of the risk of bias analysis with the Evidence Project tool was 5.88 out of 8 with a standard deviation of 1.31, and scores ranged from 3 to 7, as Table 3 shows. All the articles satisfactorily reached the items corresponding to the assessment of study design quality (items 1 and 2). Seven studies did not fulfill item 3.

In contrast, the participants’ representativeness evaluation shows more heterogeneous results. Item 4, which assessed the “random assignment of participants to the intervention”, was reached by all studies. However, the two articles did not fulfill item 5 (“random selection of participants for assessment”). Five studies did not fulfill item 6 (“follow-up rate of 80% or more”). Finally, in the equivalence of comparison groups, item 7 was fulfilled by 23, and item 8 by 25.

## 4. Discussion

This systematic review aimed to analyze which tools are the most suitable for measurable outcomes in patients with CP treated with physical therapy and other therapies. It provided an overview of evaluation procedures used in different physical therapy modalities and verification if technological advance has started to be implemented in the process. The present study identified 77 instruments to measure CP outcomes to analyze physical capacities, functionality, and quality of life adapted to different ages, but mainly designed for children and youth. In addition, according to the results, 21 of the 43 studies used 10 technology-based instruments.

Regarding the instruments used by the authors, when the focus was to analyze the patients’ level of functioning and disability, the authors did not indicate a type of instrument that contemplated all aspects of interest. Of 12 tools, the Pediatric Evaluation of Disability Inventory (PEDI) and its variances were the most cited [13,15,16,24,41]. The PEDI tool had its reliability and validity tested in another study, being compared with others instruments such as Pediatric Outcomes Data Collection Instrument (PODCI) and the Child Health Questionnaire (CHQ), which showed higher internal consistency [56]. In addition, the computer adaptive test version (PEDI-CAT) was also an outcome measure that demonstrates strong construct validity and reliability in children with CP [57].

The instruments to assess aerobic fitness, dynamic balance, and spasticity seem to be better established in the literature. To estimate aerobic capacity, the 6 min walk test (6MWT) represents the most preferred test [14,15,20,41,42,43,52,55]. The 6MWT is used in children with CP to monitor changes in functional ability, providing representative data with good reproducibility regarding aerobic capacity [58]. In order to assess dynamic balance, the authors only performed three tests and were not developed for children with CP. Time Up and Go test (TUG) was the instrument more widely used [12,15,19,27,29,41,42,43,45,51,52], probably due to the simplicity of the test administration. To measure spasticity, authors had primarily used the Modified Ashworth Scale (MAS) [25,28,38,40,52,53]; this tool shows a solid, literature-based, inter- and intra-rater agreement, exhibiting a better reliability when measuring upper rather than lower extremities [59].

Additionally, regarding gross motor function, it is possible to observe a large number of studies that used the GMFM scale, versions 88 and 66 (*n* = 20) (Table 3). Both are effective and useful as outcome measures to detect changes over time in gross motor function in children with CP undergoing physical therapy [60]. The main differences between the scales are the year of publication and the score calculation and presentation. The original version is the GMFM-88, which provides scores for five dimensions and a total score. The more recent version is called the GMFM-66, which comprises 66 items and provides only a total score. As GMFM-66 is based on an interval scale and accounts for different skills difficulties, it is favored by some authors [61].

Others physical capacities, such as muscle strength, gait, and anaerobic fitness, were also well investigated in the studies selected. The instruments preferred for measuring outcomes for each variable were the hand-held dynamometer [25,33,34,36,37,45], 3D gait analysis [24,29,44,50], and the Muscle Power Sprint Test [14,17,19,43,45]. Similarly, when the studies focused on the assessment of gait, muscle balance, and activity performance, the authors preferred technological resources. For gait analysis, the authors proposed tools based on three-dimensional analysis and videography [15,24,29,30,38,44,46,50]. For the assessment of muscle strength, several authors proposed the use of dynamometers [24,25,33,34,36,37,45,46]. Muscle activity was measured by electromyography [38], and magnetic resonance imaging [34] and ultrasonography [46,51] were proposed for the assessment of muscle structure and volume. Accelerometers in this review were used in four studies to assess activity performance [14,20,27,46], although there are also previous studies in which accelerometry was used for gait analysis [6]. In all the studies that used gait, muscle, balance, and activity performance tools, complementary tools—tests, questionnaires, or clinical observations—were also employed. None of the studies reported their results by using a single assessment tool. As in one study there were several variables, the complementary assessment tools allowed for adjusting the study’s objectives and population characteristics. None of the studies that included subjects with severe impairment (GMFCS V) used technology-based assessment tools. Instead, the variables assessed were postural control-related using tools as in [16,49]: (a) SATCo test and Chailey levels of ability; (b) motor skills [48,49]; (c) Quality of Upper Extremity Skills Test and Manual Ability Classification System; and (d) gross motor function [16,18] using PEDI and ICF-CY checklists and cognitive development. Marrades-Caballero et al., 2018 [49] utilized the Communication Function Classification System (CFC).

Of the 43 studies included, only three evaluated young adults; instead, children and adolescents were the focus of CP investigations. This fact is related to the life expectancy for patients with CP, which is influenced by many aspects, when considering the effects on the severity of physical, cognitive, and sensitive disorders [62]. In individuals with mild impairment, the survival patterns are similar to the general population; for the most severely impaired, however, the mortality by 15 years old is 50% according to the overall disability score (DISAB) [63].

According to the results of our work, the studies scoring higher (7/8) in risk of bias analysis, and those with the lowest scores (3/8) use similar tests, questionnaires, and outcomes measures. Eighty-eight percent of the articles analyzed (38 articles) scored five or more out of eight items, so we can consider them to be clinical trials and protocol studies of good quality and, therefore, outcome measures acceptable for CP use. The good quality of the studies indicates that the way the instruments were used was probably adequate to achieve the results; however, the analysis as to the adequacy of the instruments used should be done more specifically in future studies. This work covers many articles that might have relevant importance for the clinical management of CP. At the same time, we consider the variability as a limitation because there is more than one assessment of the same indicator and some articles (Table 2) that describe the instruments and methodologies, but are still in the protocol phase and lack results.

Many assessment instruments have the advantage of having different options to adapt to CP’s heterogeneous population. At the same time, they have the limitation that prevents comparison among different studies. Alternatively, we note that few studies introduce technology-based assessment tools, making it also necessary to implement these resources in people with severe impairment (GMFCS V). In the future, it will be of interest to analyze which assessment tools are more sensitive and reliable, and associate these tools with the different levels of GMFCS.

## 5. Conclusions

CP dysfunctions related to physical capacity, especially gross motor function and motor skills, have drawn the attention of physiotherapy researchers. These factors are the most affected and directly impact the functionality in daily life and the quality of life of children with CP. A large number of instruments have been used in studies to measure the outcomes of physical therapy interventions. Although there is no consensus on the best tool to evaluate most of the variables studied, this systematic review may provide an overview of instrument use by authors in the field of neurorehabilitation.

The use of gaming and technology-assisted therapies in the treatment of CP has grown in the past years, showing good results in children with different types of CP. This practice can help individuals to adhere to treatment, bringing better results in rehabilitation when compared to conventional physical therapy.

## Figures and Tables

**Table 1:** Relationship between variables and instruments for assessment of individuals with CP based on International Classification of Functioning, Disability, and Health (CIF) domains.

Component	Domain	Instrument	Authors
Body functions	b117—intellectual functions	German Bayley-II Mental Scale	Stark et al., 2016
		Conners’ Continuous Performance Test, Second Edition (CCPT)	Mak et al., 2018
		Communication Function Classification System (CFC)	Marrades-Caballero et al., 2018
	b440—respiration function	Spirometry	Choi et al., 2016
		Peak flow meter	Choi et al., 2016
	b455—exercise tolerance	6 Min Walk Test (6MWT)	Chen et al., 2016; Hilderley et al., 2016; Mitchell et al., 2016; Santos et al., 2016; Cleary et al., 2017; Peungsuwan et al., 2017; Mak et al., 2018; Schranz et al., 2018
		Submaximal treadmill test	Cleary et al., 2017
		10 m Shuttle Run Test (SRT)	Gibson et al., 2017
		10 × 5 m Sprint Test	Clutterbuck et al., 2018
	b730—muscle power function	Muscle power sprint test	Cleary et al., 2017; Gibson et al., 2017; Clutterbuck et al., 2018; Schranz et al., 2018; Kara et al., 2019
		Leg press	Kara et al., 2019
		Hand-held dynamometer	Pool et al., 2016; Kassee et al., 2017; El Shamy et al., 2018; Alhusaini et al., 2019; Inguaggiato et al., 2019; Kara et al., 2019
		Isokinetic dynamometer	Ryan et al., 2016; Damiano et al., 2017
		30 s Sit-to-Stand Test (30sSTST)	Peungsuwan et al., 2017; Mak et al., 2018
		Lateral step-up test	Mak et al., 2018
		Half-kneel to stand	Mak et al., 2018
		Standing broad jump, vertical jump, and seated throw	Clutterbuck et al., 2018
	b735—muscle tone functions	Modified Ashworth Scale (MAS)	Chen et al., 2016; Moura et al., 2016; Adar et al., 2017; El-Shamy et al., 2017; El Shamy et al., 2018; Lin et al., 2018; Mahmood et al., 2019
		Ultrasonography	Adar et al., 2017
		Comprehensive Spasticity Scale (CSS) score	Qi et al., 2017
		Tardieu Scale	Hilderley et al., 2016
	b749—muscle function, other specified and unspecified	Ultrasonography	Hosl et al., 2018
		Sit-and-reach test	Mak et al., 2018
	b755—involuntary movement reaction functions	Force plate	Lazzari et al., 2016; Gatica Rojas et al., 2017
		Good balance system	Saxena et al., 2016
		Time Up and Go (TUG)	Chen et al., 2016; Hilderley et al., 2016; Lazzari et al., 2016; Santos et al., 2016; Adar et al., 2017; Peungsuwan et al., 2017; Bjornson et al., 2018; Clutterbuck et al., 2018; Hosl et al., 2018; Schranz et al., 2018; Kara et al., 2019
		Pediatric Balance Scale (PBS)	Chen et al., 2016; Lazzari et al., 2016; Santos et al., 2016; El-gohary et al., 2017
		Functional Reach Test (FRT)	Peungsuwan et al., 2017
	b760—control of voluntary movement functions	Segmental Assessment of Trunk Control (SATCo test)	Curtis et al., 2017
		Chailey Levels of Ability	Marrades-Caballero et al., 2018
		Biodex Isokinetic Dynamometer	El-gohary et al., 2017
		Selective Control Assessment of the Lower Extremity (SCALE)	Chen et al., 2016; Pool et al., 2016; Ryan et al., 2016; Damiano et al., 2017
		Boyd and Graham’s ordinal scale	Pool et al., 2016
	b770—Gait pattern functions	3D Gait Analysis (3DGA)	Abdel-aziem and El-Basatiny, 2016; Damiano et al., 2017; Hosl et al., 2018; Gillett et al., 2019
		Gait Profile Score (GPS)	Schranz et al., 2018; Gillett et al., 2019
		Gait efficiency by Net nondimensional oxygen cost (NNcost)	Ryan et al., 2016
		Electronic walkway	Hilderley et al., 2016; Hussein et al., 2019
		Videography	Ryan et al., 2016; Hilderley et al., 2016
	b789—Movement functions, other specified and unspecified	Three dimensional analysis (3D)	Moura et al., 2016
Body structure	s770—additional musculoskeletal structures related to movement	Magnetic Resonance Imaging (MRI)	Pool et al., 2016
		Electromyography	Moura et al., 2016
		Ultrasonography	Ryan et al., 2016;
Activities and participation	d420—transferring oneself/d469—walking and moving, other specified and unspecified	Gross Motor Function Classification System	Choi et al., 2016; Hilderley et al., 2016; Ryan et al., 2016; Adar et al., 2017; Kassee et al., 2017; Clutterbuck et al., 2018; Marrades-Caballero et al., 2018; Kara et al., 2019; Mahmood et al., 2019
		Gross Motor Function Measure Challenge Module (GMFM Challenge)	Hilderley et al., 2016; Clutterbuck et al., 2018
		GMFM-88	Abdel-aziem and El-Basatiny, 2016; Adar et al., 2017; El-gohary et al., 2017; Reiffer et al., 2017; Ben-Pazi et al., 2018; Lin et al., 2018; Kara et al., 2019; Mahmood et al., 2019
		GMFM-66	Choi et al., 2016; Hilderley et al., 2016; Ryan et al., 2016; Stark et al., 2016; Santos et al., 2016; Curtis et al., 2017; Qi et al., 2017; Deutz et al., 2018; Hosl et al., 2018
		1 Min Walk Test (1MWT)	Bjornson et al., 2018; Kara et al., 2019
		10 m Walk Test (10 mWT)	Santos et al., 2016; Peungsuwan et al., 2017; Reiffer et al., 2017; Bjornson et al., 2018
		Test of Gross Motor Development-2 (TGMD-2)	Clutterbuck et al., 2018
		Peabody Developmental Motor Scales, Second Edition (PDMS-2)	El Shamy et al., 2018; Alwhaibi et al., 2020
	d445—hand and arm use	ABILHAND–kid’s questionnaire	Kassee et al., 2017
		Quality of Upper Extremity Skills Test (QUEST)	Moura et al., 2016; El-Shamy et al., 2017; Ben-Pazi et al., 2018
		Manual Ability Classification System (MACS)	Kassee et al., 2017; Kara et al., 2019; Marrades-Caballero et al., 2018
		Melbourne Assessment of Unilateral Upper Limb Function-2 (Melbourne-2)	Kassee et al., 2017
		Goal Attainment Scaling (GAS)	Gibson et al., 2017
		High Level Mobility Assessment Tool (HiMAT)	Gibson et al., 2017
		Jebsen–Taylor Hand Function Test (JTHFT)	Alhusaini et al., 2019
		Box and Block Test (BBT)	Inguaggiato et al., 2019
	d450—walk	Accelerometer	Mitchell et al., 2016; Ryan et al., 2016; Cleary et al., 2017; Bjornson et al., 2018
		Energy Expenditure Index	Schranz et al., 2018
	d920—recreation and leisure	Children’s Assessment of Participation and Enjoyment (CAPE)	Hilderley et al., 2016; Clutterbuck et al., 2018
		Preferences of Activities for Children (PAC)	Clutterbuck et al., 2018
Personal factors	Quality of life	Pediatric Quality of Life Inventory (PedsQL)-CP	Adar et al., 2017
		Cerebral Palsy Quality of Life Questionnaire for Children (CP QOL Child)	Cleary et al., 2017; Clutterbuck et al., 2018; Mak et al., 2018
		Child Health Questionnaire (CHQ 28)	Deutz et al., 2018
		KIDSCREEN-27 parental version	Hilderley et al., 2016; Deutz et al., 2018
		Cerebral Palsy Quality of Life Questionnaire for Adolescents	Mak et al., 2018

**Table 2:** Relationship between variables and instruments for assessing functioning and disability of individuals with CP cited in the studies selected.

Variable	Instrument	Authors
Functioning and disability	International Classification of Functioning, Disability and Health-Children and Youth (ICF-CY) checklist	Hsieh et al., 2016; Pool et al., 2016; Curtis et al., 2017
	28-Item Mobility Questionnaire	Mitchell et al., 2016; Mak et al., 2018
	Activity Scale for Kids (ASK)	Hilderley et al., 2016; Bjornson et al., 2018
	Assessment of Life Habits (LIFE-H)	Mitchell et al., 2016; Ryan et al., 2016; Bjornson et al., 2018
	Assessment of Motor and Process Skills (AMPS)	Comans et al., 2017
	Canadian Occupational Performance Measure (COPM)	Hilderley et al., 2016; Comans et al., 2017; Clutterbuck et al., 2018
	Functional Mobility Scale (FMS)	Clutterbuck et al., 2018
	Pediatric Evaluation of Disability Inventory (PEDI-G; PEDI; PEDI-CAT)	Hilderley et al., 2016; Stark et al., 2016; Santos et al., 2016; Curtis et al., 2017; Damiano et al., 2017
	Pediatric Outcomes Data Collection Instrument (POCCI)	Damiano et al., 2017
	Patient-Reported Outcomes Measurement Information System (PROMIS)	Bjornson et al., 2018
	Timed Stairs Test (TST)	Schranz et al., 2018
	Wee Functional Independence Measure (WeeFIM)	Adar et al., 2017

**Table 3:** Risk of bias analysis with the Evidence Project tool.

Study	Item 1	Item 2	Item 3	Item 4	Item 5	Item 6	Item 7	Item 8	Total Score
	Study Design			Participant Representativeness			Equivalence of Comparison Groups		
Abdel-aziem (2016)	Yes	Yes	Yes	Yes	No	Yes	Yes	Yes	7/8
Adar (2017)	Yes	Yes	Yes	Yes	No	Yes	Yes	Yes	7/8
Alhusaini (2019)	Yes	Yes	Yes	Yes	No	Yes	No	No	5/8
Benpazi (2018)	Yes	Yes	Yes	Yes	No	Yes	No	No	5/8
Bjornson (2018)	Yes	Yes	Yes	Yes	No	Yes	No	No	5/8
Kai Chen (2016)	Yes	Yes	Yes	Yes	No	Yes	Yes	Yes	7/8
Choi (2016)	Yes	Yes	Yes	Yes	No	Yes	Yes	Yes	7/8
Cleary (2017)	Yes	Yes	Yes	Yes	No	Yes	No	No	5/8
Clutterbuck (2018)	Yes	Yes	No	Yes	Yes	Yes	No	No	5/8
Comans (2017)	Yes	Yes	No	Yes	Yes	No	Yes	Yes	6/8
Curtis (2017)	Yes	Yes	Yes	Yes	No	Yes	Yes	Yes	7/8
Damiano (2017)	Yes	Yes	Yes	Yes	No	Yes	Yes	Yes	7/8
Deutz (2018)	Yes	Yes	Yes	Yes	No	Yes	Yes	Yes	7/8
El-gohary (2017)	Yes	Yes	Yes	Yes	No	Yes	Yes	Yes	7/8
El-shamy (2017)	Yes	Yes	Yes	Yes	No	Yes	Yes	Yes	7/8
El-shamy (2018)	Yes	Yes	Yes	Yes	No	Yes	No	No	5/8
Gatica Rojas (2017)	Yes	Yes	Yes	Yes	No	Yes	Yes	Yes	7/8
Gibson (2017)	Yes	Yes	Yes	Yes	No	Yes	No	No	5/8
Gillett (2019)	Yes	Yes	Yes	Yes	No	Yes	No	No	5/8
Hilderley (2016)	Yes	Yes	No	Yes	No	Yes	No	No	4/8
Hosl (2018)	Yes	Yes	Yes	Yes	No	Yes	No	Yes	6/8
Hsieh (2016)	Yes	Yes	Yes	Yes	No	Yes	No	No	5/8
Hussein (2019)	Yes	Yes	Yes	Yes	No	Yes	Yes	Yes	7/8
Inguaggiato (2019)	Yes	Yes	Yes	Yes	No	Yes	No	Yes	6/8
Kassee (2017)	Yes	Yes	Yes	Yes	No	Yes	No	No	5/8
Kayakara (2019)	Yes	Yes	Yes	Yes	No	Yes	Yes	Yes	7/8
Lazzari (2016)	Yes	Yes	Yes	Yes	No	Yes	Yes	Yes	7/8
Lin (2018)	Yes	Yes	Yes	Yes	No	Yes	Yes	Yes	7/8
Mahmood (2019)	Yes	Yes	Yes	Yes	No	Yes	Yes	Yes	7/8
Mak (2018)	Yes	Yes	Yes	Yes	No	Yes	No	Yes	6/8
Marrades-caballero (2018)	Yes	Yes	Yes	Yes	No	Yes	Yes	Yes	7/8
Mitchel (2016)	Yes	Yes	Yes	Yes	No	Yes	Yes	Yes	7/8
Moura (2016)	Yes	Yes	No	Yes	No	No	No	No	3/8
Peungsuwan (2017)	Yes	Yes	Yes	Yes	No	Yes	Yes	Yes	7/8
Pool (2016)	Yes	Yes	Yes	Yes	No	Yes	Yes	Yes	7/8
Qi (2017)	Yes	Yes	Yes	Yes	No	Yes	Yes	No	6/8
Reem (2020)	Yes	Yes	Yes	Yes	No	Yes	Yes	Yes	7/8
Reiffer (2017)	Yes	Yes	No	Yes	No	No	No	No	3/8
Ryan (2016)	Yes	Yes	No	Yes	No	No	No	No	3/8
Saxena (2016)	Yes	Yes	Yes	Yes	No	Yes	No	No	5/8
Schranz (2018)	Yes	Yes	Yes	Yes	No	Yes	No	No	5/8
Stark 2016	Yes	Yes	Yes	Yes	No	Yes	Yes	Yes	7/8
Villaltasantos (2019)	Yes	Yes	No	Yes	No	No	No	No	3/8

Item 1: Cohort. Item 2: Control or comparison group. Item 3: Pre- and post-intervention data. Item 4: Random assignment of participants to the intervention. Item 5: Random selection of participants for assessment. Item 6: Follow-up rate of 80% or more. Item 7: Comparison group equivalent on sociodemographics. Item 8: Comparison group equivalent at baseline on outcome measures.

## Data Availability

Not applicable.

## Supplementary Materials

The following are available online at https://www.mdpi.com/article/10.3390/jpm11070604/s1, Table S1—Formal characteristics of the selected studies and main outcomes of the interventions (*n* = 37). Table S2—Formal characteristics of the design of randomized control trials protocols included (*n* = 6).
