# Supplementary material for: Standardized Outcomes Measures in Physical Therapy Practice for Treatment and Rehabilitation of Cerebral PALSY: A Systematic Review
Source: J Pers Med. 2021 Jun 26;11(7):604. doi: 10.3390/jpm11070604 (PMC8303849; doi:10.3390/jpm11070604)
Supplement: Supplementary file 1 [file jpm-11-00604-s001.zip › jpm-1168910-supplementary.pdf]

Supplementary File Table S1. Formal characteristics of the selected studies and main outcomes of the interventions (n = 37).

Author	Country	Objectives	Sample	Type of CP	Intervention	Results
**Abdel-aziem and El-Basatiny (2016)**	Egypt	To assess the effect of backward walking training on spatiotemporal gait parameters and gross motor function measures.	n= 30 (EG: 15; CG: 15) age: 10-14 years old	Spastic hemiparesis GMFCS I-II	3 times/week for 12 w; EG: One-hour conventional physical therapy program plus 25 min. of backward walking training; CG: One hour of the same conventional physical therapy (PT) of EG plus 25 min. of forward walking training.	There was a significant improvement in step length, walking velocity, cadence, stance phase, and swing phase percentage and gross motor function measures (Dimensions D and E) of the experimental group.
**Chen at al. (2016)**	USA	To evaluate the effectiveness of a home program, which included both passive and active movement training using a portable robot.	n=41 Lab-based group (n=18); Home-based group (n=23); age: 7-18 years old.	Spastic GMFCS I-III	3 times/week for 6 w; Program: Combined passive stretching (10 min. pre and post) and active movement intervention (20 min) of one ankle joint with a portable rehabilitation robot.	Home-based and Lab-based groups showed improvements after the intervention, however there were no significant differences in the outcome measures between the two groups.
**Choi et al. (2016)**	Korea	To investigate the effect of incentive spirometer exercise (ISE) on pulmonary function and maximal phonation time (MPT).	n= 50 (EG: 25; CG: 25) age: 8-15 years	Spastic GMFCS I-IV	4 weeks EG: conventional exercise plus flow-oriented incentive spirometer which has a chamber-containing ball (10 sessions were performed daily); CG: conventional exercise (5 times/week).	There were significant improvements in pulmonary function parameters and MPT in the EG, but not in the control group. In addition, the improvements in EG were significantly greater in the experimental group than in the control group.
**Hsieh et al. (2016)**	China	To evaluate the effects of hippotherapy on body functions, activities, and participation in children with CP of various functional levels by using the ICF-CY checklist.	n=14 age: 3-8 years old.	Spastic diplegia, spastic quadriplegia, dyskinetic and hypotonic. GMFCS I, III-V	Once weekly for 12 w; Hippotherapy: warm-up (5min.), horse walking (10 min. in clockwise and 10 min. in counterclockwise), cool down (5min);	Significant improvements in ICF-CY qualifiers were found in neuromusculoskeletal and movement-related functions, mobility and major life areas and when compared with baseline.
**Lazzari et al. (2016)**	Brazil	To investigate the effects of transcranial direct current stimulation combined (tDCS) with virtual reality training on static and functional balance.	n= 20 (EG: 10; CG: 10) age: 4-12 years old	NR GMFCS I-III	5 times/week for 2 w; Virtual reality mobility training was performed using the "Your Shape: Fitness Evolved 2012" followed by active tDCS (EG) and sham tDCS (CG).	The analyses demonstrated statistically significant post intervention and follow-up effects favoring the experimental group over the control group.
**Mitchell et al. (2016)**	Australia	To test the efficacy of Mitii training on activity capacity and performance.	n=102 (EG: 51; CG: 51) age: 8-17 years old	Unilateral GMFCS I-II	20 weeks EG: 6 times/week (30 min) of web-based therapy program that combines upper-limb, visual-perceptual, and lower-limb strength training; CG: care as usual.	EG completed a mean 32.4 hours (SD 17.2) of training, associated with significant improvements in functional strength and 6MWT distance compared with the control group.

**Pool et al. (2016)**	Australia	To determine if neuromuscular electrical stimulation (NMES) applied to the ankle dorsiflexors during gait improves muscle volume and strength.	n= 32 (EG: 16; CG: 16) age: 10y8m (SD± 3y3m)	Spastic unilateral GMFCS I-II	EG: 8-weeks of Walk Aide use for at least 4 hours per day, 6 days per week. This; CG: usual or conventional care.	Treatment group demonstrated significantly increased muscle volumes and dorsiflexion strength not only when compared to their baseline values but also when compared to the control group at week 8.
**Saxena et al. (2016)**	India	To assess the feasibility of using short-term balance training with computer-based visual feedback (BTVF) and its effect on standing balance.	n= 14 (EG: 7; CG: 7) age: 5-12 years old	Spastic bilateral GMFCS II-III	EG: 4 sessions of BTVF (two such sessions/day, each session = 15 min) and usual physiotherapy (stretching exercises, functional reach activities in sitting, and fine-motor training of upper extremities); CG: usual physiotherapy.	There were no differences in the retention percentages and in any clinical outcome measure between both groups.
**Stark et al. (2016)**	Germany	To investigate the safety and feasibility of 14 weeks of home-based side-alternating whole body vibration (sWBV) training and to explore the efficacy with the Gross Motor Function Measure (GMFM-66) as change from week 0 to 14.	n=24 (Group A: 12; Group B: 12) age: 19 months (SD± 3.1)	NR GMFCS II-IV	Group A (T0 – T1): 14 weeks home-based sWBV with ten 9-minute sessions weekly (3x3 min.) The frequency of vibration was either 12 Hz or 22 Hz; Group B (T1 – T2): The same intervention.	Developmental change between T0 and T1 was similar in both groups. The intervention was feasible and safe in toddlers with CP, but was not associated with improvement in gross motor function.
**Adar et al. (2017)**	Turkey	To compare the effects of aquatic exercises and land-based exercises on spasticity, quality of life, and motor function.	n= 32 (Aquatic group: 17; Land group: 15) age: 9.7±2.7 years old.	Spastic GMFCS I-IV	5 times/week for 6 w. Aquatic group: aquatic exercise program consisted of 30 sessions in a swimming pool at 33 °C (60min); Land group: land-based exercise program consisted of 30 sessions of active range of motion exercises and stretching exercises, followed by aerobic exercise and strengthening exercise (60 min).	Both group showed significant improvements in most functional outcome measures. There were no significant differences in the percentage changes of the scores for functional outcome measures between the two groups. However, aquatic exercise produced a higher improvement in quality of life scores than the land-based exercises.
**Cleary et al. (2017)**	Australia	To estimate the effect of an aerobic training program on the cardiovascular system, physical activity, school function, participation, and quality of life of children with CP.	n= 19 (EG: ; CG:) age: 8-18 years old	NR GMFCS I-III	3 times/week over 3w (one rest day between each sessions; EG: Aerobic exercise program consisting of 10 min. on a treadmill, stationary bike, stepping machine or arm ergometer followed by 20 min. of outdoor activities such as walking, running, playing football or basketball (30min) CG: Social/art activities over the same time.	Effect sizes favored the intervention group for measures of cardiovascular performance (sub-maximal treadmill test, effect size d = 0.7; muscle power sprint test, d = 0.9) and participation (Preference for Active-Physical Activities, d = 0.6). There were no within or between group differences related to quality of life.
**Comans et al. 2017**	Australia	To estimate the cost-effectiveness of the Mitii training system for	n= 102 (EG: 51; CG: 51)	Spastic unilateral MACS I-III	20 weeks	There were significantly more responders in the training group on both the AMPS motor and

		improvements in upper limb function.	age: 8-18 years		EG: 6 times/week (30 min) of web-based therapy program that combines upper-limb, visual-perceptual, and lower-limb strength training; CG: care as usual.	process scales and the COPM performance and satisfaction scales.
**Curtis et al. 2017**	Denmark	To determine whether segmental training is more effective in improving gross motor function in children and young people with moderate-to-severe cerebral palsy than conventional physiotherapy.	n= 28 (EG: 14; CG: 14) age: 2-15 years old	NR GMFCS III-V	5 days/week over 6 months EG: Trained head and trunk control using targeted training system (30min); CG: conventional physical therapy including strength training, passive stretching, functional training, and balance and coordination training.	Segmental training was not superior to usual care in improving GMFM. Improvements in head and trunk sway were greater in the segmental training group at primary endpoint but not at follow-up.
**Damiano et al. (2017)**	USA	Quantify and compare effectiveness of a motor-assisted cycle and a novel alternative, an elliptical, to improve interlimb reciprocal coordination through intensive speed-focused leg training.	n=27 (Elliptical group: 14; Cycle group: 13) age: 5-17 years old	Spastic bilateral GMFCS I-III	5 times/week over 12 w of home-based training at a minimum 40 repetitions per minute with resistance added when speed target achieved (20min).	Ask-specific effects were similarly positive across groups, but no transfer was seen to gait or function. Training dose was low (≤20 hours) compared to intensive upper limb training recommendations and may be insufficient to produce appreciable clinical change.
**El-gohary et al. (2017)**	Saudi Arabia	To compare the effectiveness of balance training using the Biodex balance system and a conventional balance training programme on balance score and on gross motor skills.	n= 48 (EG: 24; CG: 24) age: 5-8 years old.	Spastic diplegia GMFCS Non specific	3 times/week over 3 months; EG: dynamic balance training using the Biodex balance system, in addition to traditional physical therapy programme. CG: traditional physical therapy programme in addition to postural control exercises.	Significant improvement was observed in all outcome measures of the two groups, comparing their pre- and post-treatment mean values. The results revealed a significant ($p < 0.05$) improvement in mean post-treatment values for the Biodex balance training group.
**El-Shamy (2017)**	Saudi Arabia	To examine the efficacy of Armeo® robotic therapy, compared to conventional therapy, on upper extremity function.	n= 30 (Group A: 15; Group B: 15) age: 6-8 years	Hemiplegia MACS I-III	3 times/weel over 12 w EG: Armeo robotic therapy that combines robotic assistance and virtual reality to provide a unique way to engage children in the repetitive motions required for motor learning (45min); CG: conventional therapy for the same period.	Children in the study group showed significant improvement in the mean values of all the measured variables, compared to those in the control group ($P < 0.05$).
**Gatica-Rojas et al. (2017)**	Chile	To compare the effect of Nintendo Wii balance board (Wii-therapy) and standard physiotherapy (SPT), on the performance of standing balance	n= 32 (Wii-Therapy: 16; Standard Physiotherapy: 16) age: 7-14 years old	Spastic hemiplegia and spastic diplegia. GMFCS I-II	3 sessions per week over a period of 6 weeks. Wii-Therapy: training sessions using the Wii Fit Plus with the Nintendo Wii Balance Board for 30 minutes, divided into three series; SPT: stretching, flexibility, strengthening, and	Wii-therapy was better than SPT in improving standing balance in patients with CP, but improves the balance only in spastic hemiplegic patients.

		in children and adolescents with CP.			balance exercises for 40 minutes in each session.	
**Gibson et al. (2017)**	Australia	To evaluate effects of a running intervention on running ability and participation.	n=42 (EG: 21; CG: 21) age: 12.5 y (SD ± 2.8y)	NR GMFCS I-II	EG: 2 sessions/week over 12 w of running intervention and two additional exercise sessions (at home or in a gym) per week; CG: usual care provided by their community therapist.	Statistically significant group differences at 12-weeks were found for improvements in running ability (p<0.001), and participation in the school environment (p=0.045).
**Kassee et al. (2017)**	Canada	To explore the differences between a more novel approach to rehabilitation, such as the Wii, to a more conventional therapy in the home environment.	n= 6 (Wii group: 3; Resistance training group: 3) age: 7 – 12 years old	Spastic hemiplegia GMFCS I-II	5 days a week for 6 weeks at least 40 minutes each day Wii group: Wii training to be performed at home according the patient choice (tennis, archery, swordplay, basketball, bowling, canoeing, golf and Frisbee); Resistance training group: 6 exercises to do at home with resistance bands, at an intensity of 12 repetitions per exercises, for two sets.	Descriptive statistic was used. In both groups was observed improvements in upper limb quality of movement and functional ability. Grip strength improvements were observed in 3 participants, two of which were in the resistance training group. The Wii training group reported higher compliance and more consistently positive responses to motivation and feasibility questions.
**Peungsuwan et al. (2017)**	Thailand	To investigate the effects of combined exercise training on functional performance.	n=15 (Exercise group: 8; CG: 7) age: 7-16 years old	Spastic hemiplegia or diplegia GMFCS II-III	8 weeks Exercise group: PT (1 time/week) followed by a functional exercise program that included a combined strength and endurance training program (70min; 3 times/ week); CG: conventional PT once a week (passive leg muscle stretching, upper limb activity in groups, sitting or standing ball throwing, handiwork, and a leisurely walk or playing for 1 hour during school hours).	Combined exercise training improved walking ability, functional lower limb strength, and balance in participants with cerebral palsy when compared with control group.
**Qi et al. (2017)**	China	To investigate the effect of neuromuscular electrical stimulation (NMES) combined with strengthening exercise on movement.	n= 100 (EG: 50; CG:50) age: 4-9 years old	Spastic GMFCS non specific	5 times/week for 6 w. Treatment group: 20 min. of NMES (KT-90B nerve injury treatment instrument) + strengthening exercises (crouching to stand again; weight training and navigating the stair); CG: NMES (same protocol of treatment group).	NMES combined with strengthening exercise was more effective than NMES alone in the recovery of spastic CP.
**Ben-Pazi et al. (2018)**	Israel	To investigate the impact of auditory stimulation on motor function in children with CP and disabling hypertonia.	n=18 (EG: 9; CG: 9) age: 7y5m (SD± 4y1m)	Quadriplegia, diplegia, hemiplegia GMFCS III-V	10 minutes; 4 times/week for 4 w; EG: auditory stimulation using sound frequencies in the gamma range modulated in frequency and/ or amplitude according to a	Children receiving auditory stimulation attained more goals than children who listened to music alone (p = 0.002). Upper extremity skills improved in the study group compared to

					fixed protocol; CG: Music and nature sound according to the child's preference.	controls (p = 0.006). Similar gross motor function changes were documented in both groups (p = 0.41).
**Bjornson et al. (2018)**	USA	To examine the effect of short-burst interval locomotor treadmill training (SBLTT) on walking capacity and performance.	n=12 (High frequency training: 6; Low frequency training: 6) age: 5-12 years old	Spastic diplegia GMFCS II-III	All participants received 20 sessions of SBLTT consisting of short-bursts (30 seconds) of high-speed walking intervals alternating with 30 seconds of low-to-moderate speed walking on a treadmill. High frequency training: 4weeks (5x/week); Low frequency training: 10 weeks (2x/week).	All tests related to walking capacity improved post SBLTT (p < 0.05). Walking performance increased: average strides/day (p < .001). The high-frequency group improved self-selected gait speed immediately post training compared to low-frequency group and LIFE-H recreation subscale increased significantly more for the high-frequency group than low-frequency group.
**Deutz et al. (2018)**	Germany	To investigate the effect of hippotherapy on gross motor function and quality of life.	n= 73 (Early treatment (ETG): 35; late treatment (LTG): 38) age: age: 9.1 ± 3.3 years	Spastic bilateral GMFCS II-IV	1 – 2 sessions/week during a period of 16 to 20 w. ETG: conventional physiotherapy + hippotherapy; LTG: conventional physiotherapy.	Hippotherapy shows distinct therapeutic strengths with regard to promoting upright stand and gait in children with cerebral palsy.
**El-Shamy et. (2018)**	Egypt	The purpose of this study was to investigate the effect of Wii training on hand function.	n= 40 (EG: 20; CG: 20) age: 8-12 years old	Spastic hemiplegia MACS I-III	3 times/week for 12 w. EG: Usual care plus 40 min. of Wii training program (tennis, boxing, bowling and basketball) using the Wii gaming system; CG: Usual care (1 hour of passive stretching for elbow and wrist flexors, weight-bearing exercises for the upper limbs, and protective extensor thrust).	Wii training plus usual care decreases spasticity and increases grip strength and hand function in children with hemiplegic cerebral palsy when compared with a control group.
**Hosl et al. (2018)**	Germany	To evaluate the effects of eccentric training by backward-downhill treadmill training (BDTT) and plantarflexor stretching concerning gait and muscle function.	n= 10 (EG: 5; CG: 5) age: 12 ± 4 years old.	Spastic unilateral or bilateral GMFCS I-II	3 sessions/week for two 9w. EG: BDTT with an Atlantis treadmill and a ceiling-mounted safety harness. CG: Static calf stretching program consisted of 7 exercises (5 repetitions per leg and end-range positions were held for 20 s).	BDTT can be an effective gait treatment, probably improving coordination or reducing dynamic stretch sensitivity. More intense BDTT might be necessary to further alter muscle-tendon properties. Manual static plantarflexor stretching may not be optimal in Cerebral Palsy patients with high ambulatory status.
**Lin et al. (2018)**	China	To investigate the effect of combined use of radial extracorporeal shock wave therapy (rESWT) and conventional rehabilitation	n=82 (EG: 43; CG: 39) age: 6-12 years old	Spastic GMFCS non specific	Once a week, each time for 10 min, and continued for 4 weeks. Routine rehabilitation plus rESWT protocol; CG: Physiotherapy.	The rESWT combined with rehabilitation can quickly and effectively relieve paralysis of lower extremities, reduce the tension of hamstrings and calf muscles, relieve muscle

		therapy on postoperative rehabilitation.				spasm, and rapidly improve limb function in children with spastic cerebral palsy.
**Mak et al. (2018)**	Australia	To investigate the efficacy of an embodied mindfulness-based movement programme (MiYoga), in attention and physical outcomes.	n=42 (EG: 21; CG: 21) age: 9.1 ± 3 years old.	Unilateral or Bilateral GMFCS I-III	8 weeks EG: MiYoga program based on mindfulness and mindful movement techniques based on hatha yoga principles (90min); CG: waitlist.	MiYoga can enhance attention (more attentive and consistent performance) in children with cerebral palsy. However, had no significant effect on physical functioning.
**Marrades-Caballero et al. (2018)**	Spain	To understand whether neurologic music therapy (NMT) has an impact on the functionality.	n= 18 (EG: 9; CG: 9) age: 4-18 years old	Bilateral GMFCS IV-V	32 weeks with a cross-over between groups in 16 weeks. EG (T1-T1): NMT (13 sessions) in addition to the usual physiotherapy; CG (T0-T1): physiotherapy once a week.	Significant improvements in the overall and specific "arm and hand position" as well as "activities" were observed (p<0.05) in the group which received NMT.
**Schranz et al. (2018)**	Austria	Does home-based progressive resistance (PRT) or high-intensity circuit training (HICT) improve strength, function, activity, or participation in children with cerebral palsy (CP)?	n=22 age: 8-16 years old	Spastic unilateral or bilateral GMFCS I-II	3 times weekly in both groups for 8 weeks. PRT group: trained with progressive overload; HICT group: performed as many repetitions as possible within 30-second intervals.	Both programs improved function specific to intervention. However, only the HICT group showed significant strength and participation improvements.
**Alhusaini et al. (2019)**	Saudi Arabia	To determine whether Transcutaneous electrical nerve stimulation (TENS) combined with therapeutic exercises helps to improves hand function by reducing spasticity	n= 29 (TENS group: 15; CG: 14) age: 6 – 12 years old	Hemiplegia MACS I-III	3 times weekly for 8 weeks. TENS group: Physiotherapy plus high-frequency at 90% motor threshold was activated for 30 min. with a pulse duration of 250ms and pulse rate of 100/second and biphasic square waves at the intensity 50 mA. CG: Physiotherapy (45–60 min. of active assisted and resistance movements).	The results showed a significant intergroup difference in handgrip strength over the 8-week period. The use of TENS in combination with therapeutic exercise may improve strength and hand function.
**Gillett et al. (2019)**	Australia	To evaluate the secondary effects of the functional anaerobic and strength training (FAST) intervention on gait kinematics and kinetics.	n= 17 (EG: 8; CG: 9) age: 21±4 years old	Spastic unilateral or bilateral GMFCS I-II	3 sessions/week for 12 w. EG: Combined progressive resistance and functional anaerobic training program CG: received no prescribed training and were allowed to continue with usual daily activities.	There were no between-group differences after the intervention for any kinematic or kinetic gait outcome variable.
**Hussein et al. (2019)**	Egypt	To evaluate the effect of simultaneous proprioceptive - visual training on gait parameters.	n= 30 (EG: 15; CG:15) age: 4-6 years old	Spastic diplegia GMFCS II-III	3 times/week for two months. EG: regular therapeutic exercise (1 hour) plus gait training on TekscanK-Scan Sensors (30 min); CG: regular therapeutic exercise program (1 hour; stretching and	There were significant differences after treatment in spatial parameters and temporal parameters of both groups with more improvement in study group than control one, and insignificant difference in kinetic gait parameters.

					strengthening exercises) and traditional gait training (30 min).	
**Inguaggiato et al. (2019)**	Italy	To evaluate the effect of a single anodal Transcranial Direct Current Stimulation (tDCS) application over the ipsilesional motor cortex on the unilateral gross manual function of the more affected, hemiplegic, contralesional hand.	n= 8 (EG: 5; CG: 3) age: 10-28 years old.	Unilateral MACS I-III	EG: During active tDCS, a constant current of 1.5mA was applied for 20 minutes, with a ramping period of 30 seconds at both the beginning and end of stimulation; CG: Sham tDCS was applied with the same parameters and electrode montage as active tDCS, but the current lasted only 30 seconds.	Results showed, only following the active stimulation, an immediate improvement in unimanual gross motor dexterity of hemiplegic, but not of nonhemiplegic hand. Such improvement remained stable for at least 90 minutes. Performance of both hands in Hand Grip Strength test was not modified by anodal tDCS.
**Kara et al. (2019)**	Turkey	To investigate the effects of a novel functional strength and power-training program on gait and GMF.	n=30 (EG: 15; CG: 15) age: 7-16 years old	Spastic unilateral GMFCS I MACS I-III	3 times/week for 12 w; EG: 60 min. of progressive resistance strength training exercises; CG: usual care.	Significantly greater improvements were seen in the EG for muscle power, GMF Measure E score, and 1-minute walk test, as well as for dynamic balance, 1-repetition maximum, and muscle strength.
**Mahmood et al. (2019)**	Pakistan	To evaluate the effects of traditional massage (TM) on spasticity and gross motor function	n= 75 (EG: 38; CG: 37) age: 2-10 years old	Spastic diplegia GMFCS I-III	Once daily, 5 times/week for 3 months. EG: conventional physical therapy (CPT) (30min) and TM (30min) additionally; CG: CPT.	TM can effectively reduce the spasticity, does not have harmful effects and in order to achieve better gross motor function, it should be practiced in conjunction with CPT, functional skills and task oriented approaches.
**Alwhaibi et al. (2020)**	Saudi Arabia	To study the effectiveness of visual and auditory augmented feedback using the E-link exerciser system to improve eye-hand coordination.	n= 45 (Physical Therapy: 15; Augmented biofeedback: 15; Combined group: 15) age: 8-15 years old	Spastic hemiplegia MACS I-II	3 times/week for 3 months. Physical Therapy (30min): traditional program to facilitate eye-hand coordination. Augmented biofeedback (30min): training using the E-Link Upper Limb Exerciser Combined group: same traditional physical therapy program (30 min) and training with augmented biofeedback only (30 min).	Children that received augmented biofeedback training alongside traditional physical therapy had significantly improved scores in the Visual-Motor Integration and grasping subtests compared to children that received only one intervention.

CP: cerebral palsy; GMF: gross motor function; EG: experimental group; CG: control group; ICF-CY: International Classification of Functioning, Disability and Health-Children and Youth; NR: not reported; SD: standard deviation; 6MWT: 6 minute walk test; GMFM: Gross Motor Function Measurement; GMFCS: Gross Motor Function Classification System, MACS: Manual Ability Clasification System.

Supplementary File Table S2. Formal characteristics of the design of randomized control trials protocols included (n = 6).

Author	Country	Objectives	Sample size	Type of CP	Intervention
**Hilderley et al. (2016)**	Canada	To determine the impact on gait-related gross motor skills of a gait training program using a robotic assisted gait trainer compared with a gait-related physiotherapy (PT) program.	n= 40 (Robotic assisted group: 20; PT group: 20) age: 5-12 years old	NR GMFCS II-III	2 sessions/week for 8 w. Groups was crossed over after a break of 6 weeks. Robotic group: Lokomat intervention designed as a standardized protocol (30min) and a home program component limited to basic stretching, strengthening exercises and/or walking practice that the child was on pre-study; PT group: manualized protocol created specifically by Holland Bloorview Child Development Program.
**Moura et al. (2016)**	Brazil	To perform a comparative analysis of functional training effects for the paretic upper limb with and without transcranial direct current stimulation over the primary motor cortex.	n= 34 (Group 1: anodic transcranial stimulation; Group 2: sham transcranial stimulation) age: 6-16 years old	Hemiparesis MACS I-III	Group 1: functional training of the paretic upper limb combined with anodic transcranial stimulation over the primary motor cortex; Group 2: functional training of the paretic upper limb based on manual reach with the induced constraint of the non-paretic limb and combined with sham transcranial stimulation.
**Ryan et al. (2016)**	United Kingdom	To evaluate the effect of resistance training of the ankle plantarflexors on gait efficiency, activity and participation.	n= 60 (EG: 30: CG: 30) age: 10-19 years old	Spastic GMFCS I-III	10-weeks EG: 30 sessions of resistance training focused in single-joint plantarflexor exercises; CG: usual care.
**Reiffer et al. (2017)**	Switzerland	To investigate the effectiveness of robot-assisted gait training (RAGT) on improvements of functional gait parameters.	n= 34 age: 6-18 years old	Spastic bilateral GMFCS II-IV	3 times/week for 5 w. EG: robot-assisted gait training (45 min); CG: standard treatment (1–2 sessions of physiotherapy per week and additional hippotherapy, circuit training as well as occupational therapy as necessary).
**Clutterbuck et al. (2018)**	Australia	To compare the effectiveness of a group-based, sports oriented physiotherapy intervention, Sports Stars, for ambulant school-aged children with CP, to standard care.	n=60 (Group 1: immediate intervention; Group 2: waitlist) age: 6-12 years old	All types will be included and classified by motor type/distribution. GMFCS I-II	Group 1: 8 weekly sessions (8 hours) of group-based, sports specific fundamental movement skills training (netball; soccer or softball/baseball); Group 2: standard care (neurodevelopmental therapy, context-focused therapy, strength or fitness training, or functional training).
**Santos et al. (2019)**	Brazil	To compare the clinical and functional effects of treadmill training combined with anodic transcranial direct current stimulation (atDCS) on the primary motor cortex (Cz), specifically on the area of motor cortex representation of the lower limbs, and on the cerebellum (Cb).	n= 30 (Group 1: 10; Group 2: 10; Group 3: 10) age: 7-12 years old	Spastic GMFCS I-III	5 sessions/week for 1 w. Group 1: atDCS (20min) over primary cortex and cathode over contralateral supra orbital region plus treadmill training; Group 2: atDCS (20min) over cerebellum and cathode between both supra orbital regions plus treadmill training; Group 3: Placebo tDCS (first and last 30 seconds) over primary cortex and cathode over contralateral supra orbital region plus treadmill training.
